# Individual, social and physical environmental correlates of children's active free-play: a cross-sectional study

**DOI:** 10.1186/1479-5868-7-11

**Published:** 2010-02-02

**Authors:** Jenny Veitch, Jo Salmon, Kylie Ball

**Affiliations:** 1 Centre for Physical Activity and Nutrition Research, Deakin University, 221 Burwood Hwy, Burwood, Victoria, 3125, Australia

## Abstract

**Background:**

Children's unstructured outdoor free-play (or active free-play) has the potential to make an important contribution to children's overall physical activity levels. Limited research has, however, examined physical activity in this domain. This study examined associations between individual, social and physical environmental factors and the frequency with which children play in particular outdoor locations outside school hours. This study also investigated whether the frequency of playing in outdoor locations was associated with children's overall physical activity levels.

**Methods:**

Participants including 8-9 year old children and their parents (n = 187) were recruited from a selection of primary schools of varying socioeconomic status across metropolitan Melbourne, Australia. Parents completed a survey and children's overall physical activity levels were measured by accelerometry. Regression models examined the odds of children playing in various outdoor settings according to particular correlates.

**Results:**

Inverse associations were found between preference for activities not involving physical activity, and the likelihood of children playing in the yard at home on the weekend (OR = 0.65; CI = 0.45,0.95). Positive correlates of children playing in their own street included: parental perceptions that it was safe for their child to play in their street (weekdays [OR = 6.46; CI = 2.84,14.71], weekend days [OR = 6.01; CI = 2.68,13.47]); children having many friends in their neighbourhood (OR = 2.63; CI = 1.21,5.76); and living in a cul-de-sac (weekdays [OR = 3.99; CI = 1.65,9.66], weekend days [OR = 3.49; CI = 1.49,8.16]). Positive correlates of more frequent play in the park/playground on weekdays included family going to the park together on a weekly basis on weekdays (OR = 6.8; CI = 3.4,13.6); and on weekend days (OR = 7.36; CI = 3.6,15.0). No differences in mean mins/day of moderate-vigorous physical activity were found between children in the highest and lowest tertiles for frequency of playing in particular outdoor locations.

**Conclusion:**

The presence of friends, safety issues and aspects of the built environment were reported by parents to be associated with children's active free-play in outdoor locations. Future research needs to further examine associations with time spent in active free-play and objectively-measured overall physical activity levels. It is also important to investigate strategies for developing a supportive social and physical environment that provides opportunities for children to engage in active free-play.

## Background

Opportunities for children to be active are varied and include structured activities such as organised sport or school sport; and unstructured activities such as walking or cycling to school or activity undertaken during free-play time. Children's unstructured outdoor free-play (or active free-play) represents an opportunity for children to be active and has the potential to make an important contribution to children's overall physical activity levels [1]. Compared with previous generations, children today spend less time playing outdoors within the neighbourhood [2,3], and therefore opportunities for physical activity in this domain are being missed. Considering the link between low levels of physical activity, obesity and associated chronic diseases, and the pressing need to increase children's participation in physical activity [4], it is important to investigate influences on children's active free-play.

The available evidence indicates that there are a multitude of individual, social and environmental factors that are associated with physical activity behaviour in children and that influence how children access, move and play within their neighbourhood [5]. Previous research has shown factors such as parental concerns about neighbourhood safety, availability of friends to play with, and access to interesting play areas nearby home to be important influences on children's active free-play [6,7]. There has, however, been limited research identifying where children play and in particular few studies have examined factors associated with the frequency with which children play in various outdoor locations [8]. Understanding more about children's active free-play is important as it may provide opportunities to promote physical activity among children.

Ecological models of health behaviours highlight the role of the social and physical environment in shaping individual behaviours. Using these models as the theoretical framework [9], the aim of this cross-sectional study was to investigate if specific individual, social and physical environmental factors were associated with the frequency with which children play in three specified outdoor locations. This study also examined associations between the frequency of playing in particular outdoor locations and objectively measured physical activity levels to identify whether children who play more frequently in particular outdoor locations have higher levels of overall physical activity compared with those children who do not play in those locations as frequently. Unstructured active free-play is usually engaged in more commonly among children of primary school age and therefore this age group was the focus of this research [10].

## Methods

This cross-sectional study involved the completion of a survey by 187 parents of 8-9 year old children and objective measurement of overall physical activity levels of children attending a selection of primary schools (n = 19) of varying socioeconomic status across metropolitan Melbourne, Australia. Participants were recruited from a cohort of 296 parents of 5-6 year olds who had participated in the Children's Leisure Activities Study Survey (CLASS) three years previously [10]. Of the 251 families who had indicated they were happy to take part in further research, 191 (76%) agreed to participate in this study. Upon receiving permission from participants and their child's school, each of the families were sent a parent survey to complete and were asked to return the survey to the school on the day the research team visited the school to fit the child with an accelerometer to measure their physical activity levels. The Deakin University Ethics committee, the Department of Education and Training Victoria and the Catholic Education office granted ethical approval to conduct the study.

### Items assessing frequency with which children play in particular outdoor locations

Survey items required parents to report how often their child played in the yard at home, their own street/court/footpath, and the park/playground outside school hours on weekdays and on weekend days during a typical week. These three locations were chosen as previous research showed that they were the places where children engaged in active free-play most often [7]. Responses to weekday items were marked on a five-point scale ranging from never/rarely to five days per week; and for the weekend items, on a six-point scale ranging from never/rarely to every Saturday and Sunday. In order to examine factors associated with frequency of play in different locations, responses were grouped into two categories for each item; the top third of responses (i.e., highest tertile), and the remaining responses (i.e., lowest and second tertiles).The sample size did not permit all three tertile categories to be retained, and these tertile divisions were chosen as they enabled the participants who played most frequently at particular locations (i.e. were in the top third) to be identified. The highest tertiles for these three locations were: child played in the yard at home five weekdays per week (for the weekday measure), and every Saturday and Sunday (for weekends); child played in own street/court/footpath on at least 1-2 weekdays per week (weekday measure) and on at least one day each weekend; and child played in the park/playground on at least 1-2 days per week (weekday measure) and at least one day each weekend. Two-week test-retest reliability of these items was examined among a separate convenience sample of 53 parents of 8-12 year-olds. Intra-class correlation coefficients (ICC) indicated that all items showed at least moderate reliability (ICC values ranged from 0.58-0.82) [11].

### Items examining influences on children's frequency of playing in various outdoor locations

Based on socioecological models of health [12], 16 selected items examined individual, social and environmental correlates of children's use of these outdoor locations (Table 1). Two-week test-retest reliability from 53 parents of children indicated that percent agreement was acceptable for all items (≥ 77%).

**Table 1:** Distribution, percent agreement and internal reliability of correlates of children's active free-play

		n (%)		mean (SD)		Reliability (% agreement/ICC)
**Individual Factors**						
Parent is married or in a de-facto relationship		161 (87)				-
						
Parents' education level						
<12 yrs school		35 (19)				-
12 yrs/trade		55 (30)				
University		95 (51)				
Parent does not work full-time		142 (80)				-
Number of siblings, mean (SD)				1.45 (0.96)		-
Child prefers to do things that do not involve physical activity, mean (SD)				0.53 (0.87)		ICC = 0.73
						
**Social Environmental Factors**						
Parents have time to transport their child to activities		157 (84)				92
Parents believe there is high a crime rate in their neighbourhood		19 (10)				96
Parents believe it is safe for their child to play in the street outside their house		87 (48)				77
Stranger danger is a concern		149 (81)				79
Child has many friends in their neighbourhood		98 (54)				89
Lots of children play or hang out in their street		47 (26)				81
Child's family goes to the park together at least once/week		70 (38)				92
						
**Physical Environmental Factors**						
Child's home has no yard or very small yard		20 (11)				100
Child lives in main arterial or busy through road		55 (30)				89
Child lives in cul-de-sac		51 (28)				96
Parents are satisfied with the quality of parks/playgrounds in their neighbourhood, mean (SD)				1.38 (0.87)		ICC = 0.81

### Individual factors

Parents reported their marital status, highest level of education, employment status, and the number of other children aged under 18 years (not including the child in the study) currently living in their house. Parents also indicated how much they agreed or disagreed with the following statements as reasons for their child not doing more physical activity than he/she already does: "There are other things my child enjoys doing more"; "My child just doesn't enjoy being physically active"; and "My child would prefer to watch TV or play electronic games". Responses were collapsed into two categories: agree (strongly agree or agree) or disagree (neither, disagree, strongly disagree, or don't know). These three items were combined to form one item on child preferences, "Child prefers to do things that do not involve physical activity" with results presented as a summed score ranging from 0-3, with a higher score indicating a greater level of agreement (Cronbach's alpha = 0.67).

### Social environmental factors

Parents were asked how much they agreed or disagreed with the statements: "I do not have enough time to transport my child to activities"; "There is a high crime rate in my neighbourhood"; "It is safe for my child to play or hang out in the street outside our house"; "Stranger danger is a concern of mine"; "My child has many friends in this neighbourhood"; and "Lots of children play or hang out in the street outside our house". Responses were collapsed into two categories: agree (strongly agree or agree) or disagree (neither, disagree, strongly disagree, or don't know). Parents were also asked to report how often "As a family we go to the park", with response options collapsed to: at least once per week; or less than once per week.

### Physical environmental factors

Parents were asked about their yard size with responses collapsed into two categories: no yard or small yard (e.g. unit), or medium or large yard (standard block of land or 1/4 acre or more). They also reported whether they lived on a main arterial or busy throughway for motor vehicles; and whether they lived on a cul-de-sac, court or no-through road. Finally, parents indicated how much they agreed or disagreed with the two statements "I am satisfied with the quality of parks in my neighbourhood" and "I am satisfied with the quality of playgrounds in my neighbourhood". Responses were collapsed into two categories: agree (strongly agree or agree) or disagree (neither, disagree, strongly disagree, or don't know). These two items were combined to form a single item, 'I am satisfied with the quality of parks/playgrounds in my neighbourhood' with results presented as a summed score ranging from 0-2, with a higher score indicating a greater level of agreement (Cronbach's alpha = 0.87). Access to parks was not measured.

### Children's objectively assessed physical activity

Children were asked to wear an accelerometer (Manufacturing Technologies, Inc [MTI] Model 7164; Actigraph, Inc, Florida, USA) attached to an elasticised belt at hip-level for eight consecutive days, removing it only for sleeping, showering or swimming [10]. Movement counts were recorded in 1-minute epochs. Accelerometer data files were downloaded and entered into a data-reduction program. Data were excluded for any day on which the total movement counts recorded were less than 10,000 counts (suggesting the accelerometer had not been worn as requested) or greater than 20 million counts (suggesting the accelerometer had malfunctioned) [10,13]. Only data from children with complete accelerometry data for at least four days (including at least one weekend day) were included in the analyses (n = 173). Mean minutes per day spent in physical activity of moderate-to-vigorous intensity (MVPA ≥ 3 METs) was calculated for four specific periods on weekdays - before school (i.e. 6 am to first school bell); after school (i.e. last school bell to 6 pm); evening (i.e. 6-9 pm); and outside school hours (i.e. combination of the 3 periods from 6 am-9 pm excluding school hours) - and all day on weekend days. For the weekday data, average minutes of MVPA during the after-school period was used in analyses, as this represents the time of the day when children are most likely to be engaging in active free-play [14]. For the weekend days, data for the whole day were included and the average minutes per weekend day spent in MVPA was calculated.

Statistical analyses were conducted using SPSS version 14.0 statistical software.

Unadjusted logistic regression models were performed to examine the odds of children playing in various settings on weekdays and weekend days (upper tertile) according to particular individual, social and physical environmental correlates. Variables that were significantly associated (p < 0.05) bivariably were then included in a multivariable logistic regression model. Prior to performing this multivariable analysis, collinearity between independent variables was checked by performing Pearson's correlations. When collinearity was observed, the variable that was not as strongly associated with the outcome bivariably was excluded in the multivariable model. A correlation of *r *> 0.7 is considered to be an indication of collinearity [15]; however, for these analyses a more conservative value of *r *> 0.4 was used. Only one variable was significantly bivariably associated with playing in the yard at home, therefore multivariable logistic regression was not performed for this outcome. Using equations from Hsieh et al. [16], sample size calculations for logistic regression of a binary dependent variable using normally distributed independent variables, at 80% power and a 0.05 significance level and assuming an event rate at mean of X = 0.5 and odds increase of 1.6 per unit increase in X, require a sample size of 142.

## Results

### Participants

One-hundred-and-eighty-seven parents/carers (90% were mothers) completed the survey on behalf of their child. The mean age of the survey respondents was 40 years with a range of 27-56 years. Data were analysed for 187 children (53% boys) with mean age 9.1 years (SD = 0.4).

### Frequency of playing in outdoor locations

As shown in Table 2, the yard at home was the location where parents reported children played most often, with over one-third of the sample playing there five days per week and almost two-thirds playing there every Saturday and Sunday.

**Table 2:** Distribution of active free-play frequency for each outdoor location

	Yard at home n (%)	Own street n (%)	Park/playground n (%)
**Weekdays**			
Never/rarely	4 (2.2)	72 (39.3)	38 (20.5)
<once/week	11 (6.0)	39 (21.3)	83 (44.9)
1-2 days/week	40 (21.7)	31 (16.9)^3^	48 (25.9)^4^
3-4 days/week	64 (34.8)	25 (13.7)	6.5 (12)
5 days/week	65 (35.3)^1^	16 (8.7)	2.2 (4)
			
**Weekend days**			
Never/rarely	5 (2.7)	64 (36.6)	20 (10.8)
<1 weekend/month	2 (1.1)	20 (10.8)	38 (20.5)
1 weekend/month	2 (1.1)	15 (8.1)	38 (20.5)
2 weekends/month	13 (7.1)	17 (9.2)	30 (16.2)
1 day every weekend	45 (24.7)	32 (17.3)^5^	43 (23.2)^6^
Every Saturday and Sunday	115 (63.2)^2^	37 (20.0)	16 (8.6)

^1^Highest tertile for play in yard at home on weekdays = 5 days per week

^2^Highest tertile for play in yard at home on weekend days = every Saturday and Sunday

^3,4^Highest tertile for play in own street and park/playground on weekdays = ≥ 1-2 days per week

^5,6 ^Highest tertile for play in own street and park/playground on weekend days = ≥ 1 day every weekend

### Associations with playing in particular outdoor locations

#### Playing in the yard at home

According to bivariable analysis, parents who reported a high crime rate in their neighbourhood were also almost three times as likely to report that their child played in their yard five weekdays per week compared with children whose parents did not report that they believed that there was a high level of crime in their neighbourhood (Table 3). For weekend days, multivariable analysis showed that with each unit increase in preference for activities not involving physical activity, children were 35% less likely to play in the yard at home every Saturday and Sunday.

**Table 3:** Odds of being in the upper tertile for playing in yard at home

		Yard at Home		
		**Weekdays**	**Weekend Days**	

		**Unadjusted OR** **(95%CI)**	**Unadjusted OR** **(95%CI)**	**Adjusted OR†** **(95%CI)**

**Individual Factors**				
Parent is married or in a de-facto relationship (referent = single parent)		1.12 (0.45, 2.77)	1.53 (0.64, 3.63)	-
Parents' education level				
<12 yrs school (referent)		1.0	1.0	
12 yrs/trade		1.69 (0.69, 4.12)	1.61 (0.65, 4.01)	-
University		1.06 (0.46, 2.44)	0.88 (0.39, 1.97)	-
Parent does not work full-time (referent = parent works full time)		1.18 (0.53, 2.60)	1.41 (0.66, 2.99)	-
Number of siblings (range:0-6)		1.16 (0.85, 1.58)	1.50 (1.04, 2.15)*	1.35 (0.91, 2.0)
Child prefers to do things that do not involve physical activity (range 0-3)		0.75 (0.51, 1.09)	0.57 (0.39, 0.81)**	0.65 (0.45, 0.95)**
				
**Social Environmental Factors**				
Parents have time to transport their child to activities (referent = parents do not have time)		1.25 (0.54, 2.95)	1.06 (0.47, 2.40)	-
Parents believe there is a high crime rate in their neighbourhood (referent = disagree)		2.78 (1.06, 7.30)*	3.39 (0.95, 12.12)	-
Parents believe it is safe for their child to play in the street outside their house (referent = disagree)		0.99 (0.54, 1.82)	1.20 (0.65, 2.21)	-
Stranger danger is a concern (referent = disagree)		1.14 (0.51, 2.52)	2.32 (1.09, 4.94)*	2.17 (0.98, 4.81)
Child has many friends in their neighbourhood (referent = disagree)		1.31 (0.71, 2.43)	1.98 (1.07, 3.65)*	1.59 (0.83, 3.05)
Lots of children play or hang out in their street (referent = disagree)		0.94 (0.47, 1.90)	1.75 (0.85, 3.63)	-
Child's family goes to the park together at least once/week (referent = disagree)		1.39 (0.75, 2.59)	1.64 (0.87, 3.10)	-
				
**Physical Environmental Factors**				
Child's home has no yard or very small yard (referent = home has medium or large sized yard)		0.85 (0.31, 2.36)	0.85 (0.33, 2.21)	-
Child lives in main arterial or busy through road (referent = does not live in main arterial road)		1.81 (0.95, 3.47)	1.08 (0.56, 2.10)	**-**
Child lives in cul-de-sac (referent = does not live in cul-de-sac)		0.77 (0.39, 1.54)	1.58 (0.79, 3.17)	
Parents are satisfied with the quality of parks/playgrounds in their neighbourhood (range 0-2)		0.89 (0.63, 1.26)	1.04 (0.73, 1.47)	**-**

†adjusted for all significant variables in bivariable analyses

*p < 0.05, **p < 0.01

#### Playing in child's own street/court/footpath

Multivariable analyses showed that parents who reported it was safe for their child to play in the street outside their house were also 6.5 times as likely to report that their child played in their street/court/footpath on at least 1-2 weekdays. If they lived in a cul-de-sac, they were four times as likely to report that their child played in the street/court/footpath on at least 1-2 weekdays (Table 4). For weekend days; parents who reported that they believed it was safe for their child to play in the street outside their house were also six times more likely to report that their child played in their street/court/footpath on at least one day per weekend, parents who reported that their child had many friends in the neighbourhood were also more than 2.5 times as likely to report that their child played in their street/court/footpath on at least one day per weekend, and parents who reported that they lived in a cul-de-sac were 3.5 times as likely to report that their child played in their street/court/footpath on at least one day per weekend.

**Table 4:** Odds of being in the upper tertile for playing in own street/court/footpath

		Playing in own street/court/footpath			
		**Weekdays**		**Weekend days**	

		**Unadjusted OR** **(95%CI)**	**Adjusted OR†** **(95%CI)**	**Unadjusted OR** **(95%CI)**	**Adjusted OR†** **(95%CI)**

**Individual Factors**					
Parent is married or in a de-facto relationship (referent = single parent)		1.15 (0.46, 2.68)	-	1.23 (0.49, 3.05)	-
Parents' education level					
<12 yrs school (referent)		1.0		1.0	
12 yrs/trade		0.89 (0.38, 2.10)	-	0.82 (0.35, 1.95)	-
University		0.83 (0.38, 1.84)	-	0.73 (0.33, 1.62)	-
Parent does not work full-time (referent = parent works full time)		1.28 (0.59, 2.78)	-	0.90 (0.42, 1.93)	-
Number of siblings (range:0-6)		1.05 (0.77, 1.42)	-	0.99 (0.73, 1.35)	-
Child prefers to do things that do not involve physical activity (range 0-3)		0.79 (0.55, 1.14)	-	0.62 (0.42, 0.93)	-
					
**Social Environmental Factors**					
Parents have time to transport their child to activities (referent = parents do not have time)		0.90 (0.40, 2.03)	-	0.58 (0.26, 1.30)	-
Parents believe there is a high crime rate in their neighbourhood (referent = disagree)		1.41 (0.55, 3.67)	-	1.56 (0.60, 4.05)	-
Parents believe it is safe for their child to play in the street outside their house (referent = disagree)		10.4 (5.06, 21.39)***	6.46 (2.84, 14.71)***	10.07 (4.86, 20.87)***	6.01 (2.68, 13.47)***
Stranger danger is a concern (referent = disagree)		1.32 (0.59, 2.94)	-	1.54 (0.72, 3.53)	-
Child has many friends in their neighbourhood (referent = disagree)		2.94 (1.57, 5.52)***	2.02 (0.93, 4.40)	3.42(1.79, 6.53)***	2.63 (1.21, 5.76)**
Lots of children play or hang out in their street (referent = disagree)		7.78 (3.64, 16.62)***	Omitted due to collinearity	6.67 (3.20, 13.90)***	Omitted due to collinearity
Child's family goes to the park together at least once/week (referent = disagree)		1.39 (0.76, 2.50)	-	1.27 (0.69, 2.35)	-
				
**Physical Environmental Factors**					
Child's home has no yard or very small yard (referent = home has medium or large sized yard)		0.83 (3.1, 2.19)	-	0.67 (0.25, 1.84)	-
Child lives in main arterial or busy through road (referent = does not live in main arterial road)		0.38 (0.19, 0.77)**	0.84 (0.32, 2.21)	0.19 (0.08, 0.44)***	0.42 (0.16, 1.12)
Child lives in cul-de-sac (referent = does not live in cul-de-sac)		6.33 (3.10, 12.94)***	3.99 (1.65, 9.66)**	7.72 (3.54, 14.93)***	3.49 (1.49, 8.16)**
Parents are satisfied with the quality of parks/playgrounds in their neighbourhood (range 0-2)		1.01 (0.72, 1.43)	**-**	1.10 (0.77, 1.56)	**-**

†adjusted for all significant variables in bivariable analyses

**p < 0.01, ***p < 0.001

#### Playing in the park/playground

Parents who reported that their family goes to the park together at least once per week were also approximately seven times as likely to report that their child played in the park/playground on at least 1-2 weekdays per week and on at least one day per weekend compared with children whose parents reported that their family did not go to the park together on a weekly basis (Table 5).

**Table 5:** Odds of being in the upper tertile for playing in the park/playground

		Park/Playground			
		**Weekdays**		**Weekend days**	

		**Unadjusted OR** **(95%CI)**	**Adjusted OR†** **(95%CI)**	**Unadjusted OR** **(95%CI)**	**Adjusted OR†** **(95%CI)**

**Individual Factors**					
Parent is married or in a de-facto relationship (referent = single parent)		0.87 (0.36, 2.12)	-	1.17 (0.46, 2.99)	-
Parents' education level					
<12 yrs school (referent)		1.0		1.0	
12 yrs/trade		1.25 (0.51, 3.07)	-	0.42 (0.17, 1.06)	-
University		1.20 (0.52, 2.75)	-	0.79 (0.35, 1.75)	-
Parent does not work full-time (referent = parent works full time)		1.37 (0.61, 3.09)	-	1.06 (0.48, 2.34)	-
Number of siblings (range:0-6)		1.09 (0.80, 1.49)	-	0.87 (0.62, 1.22)	-
Child prefers to do things that do not involve physical activity (range 0-3)		0.76 (0.52, 1.12)	-	0.60 (0.39, 0.93)	-
					
**Social Environmental Factors**					
Parents have time to transport their child to activities (referent = parents do not have time)		2.92 (1.06, 8.07)*	1.7 (0.6, 5.2)	2.54 (0.92, 7.04)	-
Parents believe there is a high crime rate in their neighbourhood (referent = disagree)		1.13 (0.42, 3.02)	-	2.61 (0.99, 6.82)	-
Parents believe it is safe for their child to play in the street outside their house (referent = disagree)		1.73 (0.93, 3.24)	-	1.43 (0.76, 2.68)	-
Stranger danger is a concern (referent = disagree)		0.95 (0.44, 2.08)	-	1.40 (0.61, 3.23)	**-**
Child has many friends in their neighbourhood (referent = disagree)		1.41 (0.76, 2.62)	**-**	1.39 (0.74, 2.60)	**-**
Lots of children play or hang out in their street (referent = disagree)		0.96 (0.47, 1.92)	**-**	0.57 (0.26, 1.21)	**-**
Child's family goes to the park together at least once/week (referent = disagree)		7.37 (3.70, 14.49) ***	6.8 (3.4, 13.6) ***	**8.38 (4.15, 16.94)**	7.36 (3.6, 15.0) ***
					
**Physical Environmental Factors**					
Child's home has no yard or very small yard (referent = home has medium or large sized yard)		2.11 (0.83, 5.40)	-	1.94 (0.76, 4.99)	-
Child lives in main arterial or busy through road (referent = does not live in main arterial road)		1.70 (0.89, 3.27)	**-**	1.03 (0.53, 2.03)	**-**
Child lives in cul-de-sac (referent = does not live in cul-de-sac)		1.03 (0.53, 2.03)		0.49 (0.23, 1.04)	**-**
Parents are satisfied with the quality of parks/playgrounds in their neighbourhood (range 0-2)		1.43 (0.98, 2.07)	**-**	1.86 (1.23, 2.83)**	1.49 (0.94, 2.35)

†adjusted for all significant variables in bivariable analyses

*p < 0.05, **p < 0.01, ***p < 0.001

### Associations between frequency of playing in certain outdoor locations and children's after-school and weekend physical activity

The mean number of minutes children spent per day in MVPA from the end of school until 6 pm on weekdays was 44.2 ± 15.1 minutes and on weekend days was 163.7 ± 81.0 minutes (Table 6). No significant differences in mean mins/day MVPA were revealed between children in the highest versus the bottom two tertiles for frequency of playing in their yard at home, their own street/court/footpath, or the park/playground.

**Table 6:** Mean minutes per day MVPA on weekdays and weekend days by frequency of playing in certain outdoor locations

		Mean minutes (SD) per day MVPA on weekdays (3.30-6 pm)	Mean minutes (SD) per day MVPA on weekend days
Yard at home			
- highest tertile		43.9 (13.6)	168.4 (82.17)
- Lowest and second tertile		44.6 (15.9)	154.3 (80.67)
Own street/court/footpath			
- highest tertile		45.98 (15.15)	174.4 (89.52)
- Lowest and second tertile		43.2 (15.03)	157.7 (75.49)
Park/playground			
- highest tertile		46.6 (14.45)	158.6 (88.66)
- Lowest and second tertile		43.0 (15.29)	166.5 (77.61)

## Discussion

This study identified that individual factors and aspects of the social and physical neighbourhood environment were associated with children's participation in active free-play in particular outdoor locations. The yard at home was the location where parents reported that their child played most often on both weekdays and weekend days, therefore, it seems plausible that finding ways to optimise children's active free-play in this setting may be an important aim of future research.

Consistent with previous studies that have shown individual physical activity preferences to be associated with children's overall physical activity [8,17], this study showed that parents who reported that their child preferred to spend time engaged in activities that did not involve physical activity were also less likely to report that their child play in the yard at home on the weekend. This variable relating to child's preferences was the only individual variable that was associated in the multivariable analysis with playing in any location and was only associated with playing in the yard at home and not in the child's street or local park. Although research among both adults [18] and youth [19] suggests that physical activity and sedentary behaviours can co-exist with each other, identifying and promoting physical activities that children enjoy and prefer to do may increase frequency of playing outdoors.

A number of social environmental factors were found to be associated with children's active free-play. A plausible explanation for the finding that children whose parents perceive a high crime rate play more in the yard, may be that these parents consider the yard a relatively safe environment and encourage play in the yard at home instead of other outdoor locations such as play in the street or park. This could be considered encouraging as at least the children are still playing outdoors, and is consistent with other cross-sectional studies that have suggested that parents' perceptions of neighbourhood crime may influence children's outdoor play [20].

The importance of social networks and friends to activities performed outside school hours has been identified previously [7,21,22]. This study found that parents who reported that their child had many friends in their neighbourhood were more also likely to report that their child played more regularly in their own street/court/footpath. This suggests that in order to increase children's active free-play it may be important to develop and foster social networks within the neighbourhood so that families and children can establish links for active free-play. This may involve developing community family days or other social events where families have opportunities to interact.

Previous cross-sectional studies have reported mixed results about correlations between neighbourhood safety and children's physical activity [23,24]. In the current study, parents' perceptions that it was safe for their child to play in the street outside their house was positively associated with parents' reporting that their child played in their own street/court/footpath more regularly on both weekdays and weekend days. This study did not measure real safety and therefore it is not possible to determine if there are gaps between real and perceived safety issues. Increasing real safety within the neighbourhood is likely to have positive implications for children's active free-play. In order to maximise opportunities for children to play in their street and other outdoor places, it may be important to develop education campaigns for parents about real versus perceived risks relating to children's active free-play and provide children with additional knowledge and skills for playing safely in their neighbourhood.

Whilst previous review papers have shown conflicting results for studies assessing street connectivity and physical activity among children [5], the findings from the present study indicate that living in a court/cul-de-sac was positively associated with play in the child's street. Literature regarding adult physical activity suggests that connecting streets and through-roads are important for promoting walking among adults [25]; however, in this study connecting or through-roads were inversely bivariably associated with the child playing in their own street/court/footpath. It is also possible that connecting streets may be important for children's active transport, but not so conducive to active free-play. It may be necessary to design street networks that provide the safety of a cul-de-sac to promote children's active free-play whilst also allowing connectivity to encourage walking among adults. Other alternatives may include providing connections between streets that are accessible by foot rather than vehicles and limiting through traffic in specified neighbourhood streets during after-school hours.

Parents who reported visiting parks as a family were more likely to report that their child played in the park/playground on a regular basis. Park use by families may be increased if parks are easily accessible, located close to home and provide facilities that are appealing to children of all ages [7,8]. Further studies that investigate issues surrounding playground facilities and involve children in the design process may encourage use of local parks by children and families. It is also possible that parental safety concerns about park use may be reduced if children visit parks that are located close to home [7,8].

This study showed that the frequency with which children played in the specified outdoor locations was not associated with overall objectively-assessed physical activity levels. This lack of association may be due to the non-specificity of the objective physical activity measure. That is, children may have high levels of physical activity because they were participating in other forms of physical activity such as organised sport. It is also possible that all children did not have equal amounts of free-time in the after-school period to engage in physical activity and this could potentially have an impact on the results. Finally, it is important to recognise that only the frequency of playing in particular outdoor locations was measured and future studies of active-play that measure time spent playing in each location may show associations with overall physical activity levels. Further investigation is warranted as it is probable that active free-play has the potential to make an important contribution to children's overall physical activity; however, no other Australian studies have assessed physical activity specifically in this domain.

The consideration of influences at the individual, social environmental and physical environmental level was a strength of the study design; as was the test re-test reliability of the outcome and predictor variables and the objective measure of total physical activity. Limitations include the use of a parent-report survey which relied on parents answering the survey honestly and accurately and the cross-sectional design which prevents inferences about causality. Although power calculations suggest the study was adequately powered to detect significant associations, there were smaller numbers of participants in certain categories which may have resulted in insufficient power to detect some associations between variables.

## Conclusion

Acknowledging the study limitations, the findings from this study have important implications for children's physical activity promotion and future research. Although the frequency with which children played in outdoor locations was not found to be associated with overall objective physical activity levels, aspects of the neighbourhood environment were shown to be associated with parental reports of children's active free-play. This study suggests that in order to increase children's active free-play it may be important to identify children's preferences for activities involving physical activity and encourage social networks within the neighbourhood so that families and children can establish links for active free-play. Developing education campaigns for parents about neighbourhood safety and providing children with additional skills for playing safely in their neighbourhood may maximise opportunities for children to play in their street and other outdoor places. Finally, to promote children's play, it may be necessary to design street networks that include a cul-de-sac and provide parks and playgrounds that are easily accessible and appealing to families.

Considering that active free-play provides opportunities for children to be physically active, it is important that future studies further examine associations with time spent in this domain and objectively-measured overall physical activity levels. Identifying ways to develop a neighbourhood environment that provides opportunities for children to engage in active free-play is also warranted.

## Competing interests

The authors declare that they have no competing interests.

## Authors' contributions

JV conceived the current study, completed the data analysis, and drafted the manuscript. JS and KB helped with data analysis and to draft and revise the manuscript.

All authors have read and approved the final manuscript.
